# Central Asia's Hidden Burden of Neglected Tropical Diseases

**DOI:** 10.1371/journal.pntd.0001224

**Published:** 2011-09-27

**Authors:** Peter J. Hotez, Ken Alibek

**Affiliations:** 1 Sabin Vaccine Institute and the National School of Tropical Medicine at Baylor College of Medicine, and Texas Children's Hospital, Houston, Texas, United States of America; 2 Nazarbayev University, Astana, Kazakhstan

The neglected tropical diseases (NTDs) are the most common infections of the world's poorest people living in developing countries [1]–[7]. They are mostly comprised of chronic parasitic and related infections, with the most common NTDs represented by the soil-transmitted helminthiases, schistosomiasis, lymphatic filariasis, onchocerciasis, and trachoma [1]. Among their common features, the NTDs result in prolonged periods of disability and actually help to promote poverty through their long-standing effects on child development and worker productivity [2]. It is not commonly appreciated that the NTDs are widespread throughout Central Asia where they are also a major determinant of poverty [8]. The five mostly landlocked Central Asian countries—Kazakhstan, Kyrgyzstan, Tajikistan, Turkmenistan, and Uzbekistan (Figure 1)—were established upon the breakup of the former Soviet Union in 1991. They are also linked in history as a vital crossroads (“the Silk Road”) between Asia and Europe and by a common geography comprised of a desert and piedmont region [9]. The five nations have a combined population of 60 million people, with three of them—Kyrgyzstan, Tajikstan, and Uzbekistan—exhibiting a Human Development Index (HDI) that is ranked below 100, whichis more or less equivalent to nations such as Guatemala, India, Indonesia, and South Africa [10].

**Figure 1:**
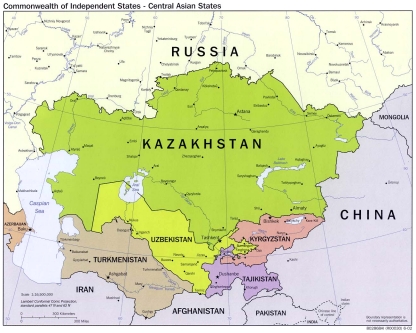
Commonwealth of Independent States - Central Asian States. Courtesy of the University of Texas Libraries, The University of Texas at Austin. Map available at: http://www.lib.utexas.edu/maps/commonwealth/central_asian_common_2002.jpg.

During the Soviet era in the 20th century, some gains were made in parasite and NTD control. For instance, in Uzbekistan a number of NTDs were either eradicated or eliminated as a public health problem, including dracunculiasis in 1931, urban cutaneous leishmaniasis in 1950, malaria in 1960, visceral leishmaniasis in 1968, and hookworm infection in 1974 [11]. However, following the 1991 breakup of the Soviet Union, public infrastructures and services deteriorated in many areas of Central Asia, and breakdowns in health care and preventive services ensued [12]. Of particular relevance to zoonotic NTDs and according to Torgerson et al., the Soviet breakup also meant that large mechanized slaughterhouses were closed, leaving livestock production in the hands of small farms and unsupervised homes, and largely without veterinary inspection [13], [14]. Together with increases in pet and security dogs, veterinary public health was compromised, with a resultant re-emergence of several important NTDs, including echinococcosis and possibly toxocariasis [13]–. As a result, today several NTDs either remain widespread in Central Asia or may have even increased in prevalence over the last two decades. They include the soil-transmitted helminth infections, food-borne and zoonotic parasitic infections, and vector-borne protozoan infections (Table 1).

**Table 1:** Selected Neglected Tropical Diseases in Central Asia.

Disease	Prevalence	Country Where Measured	Reference
**Intestinal helminth infections**			
Ascariasis	23%	Kyrgyz Republic (Osh oblast)	[12]
Enterobiasis	19%	Kyrgyz Republic (Osh oblast)	[12]
Hymenolepiasis	4%	Kyrgyz Republic (Osh oblast)	[12]
**Zoonotic helminth infections**			
Cystic echinococcosis	Up to 30 cases per 100,000 (1% of population)	Kazakhstan, Krgyz Republic, Tajiskistan, Turkmenistan, Uzbekistan	[13], [15]
Opisthorchiasis	1.2 million cases	Kazakhstan, Belarus, Ukraine, Western Siberia	[27]
Fascioliasis	2%	Kyryz Republic (Osh oblast)	[12]
Toxocariasis	11%	Kazakhstan (rural Eastern)	[15]
**Vector-borne protozoan infections**			
Leishmaniasis (cutaneous *L. major*; visceral *L. infantum*)	Not determined		[35]–[37]
Malaria	Number of probable and confirmed:	Number of suspected cases:	[39]
	Kyrgyzstan 4	Kyrgyzstan 33,983	
	Tajikistan 165	Tajikistan 165,266	
	Turkmenistan 0	Turkmenistan 94,237	
	Uzbekistan 4	Uzbekistan 916,839	
**Other protozoan infections**			
Toxoplasmosis	16% seroprevalence	Kazakhstan (rural eastern)	[15]

## Intestinal Helminth Infections

The major soil-transmitted helminth infections in Central Asia include ascariasis and enterobiasis. Overall, these nematode infections are understudied in Central Asia. The most recent complete assessment of intestinal helminth infections was provided recently by Steinmann et al., who conducted a cross-sectional study of 1,262 children among 51 rural primary schools in southwestern Kyrgyzstan [12]. It was found that most of the children had at least one intestinal helminth infection, led by ascariasis (23%), enterobiasis (19%), and hymenolepiasis (4%). Noted among the risk factors for soil-transmitted helminth infections were the absence of sanitation (ascariasis), and poverty and sharing of beds (enterobiasis). In contrast, the availability of tap water and washing of raw vegetables mitigated the risk of infection [12]. In Uzbekistan, these infections have been responsible for losses in economic productivity [16]. Steinmann et al. make the case that Kyrgyzstan (and possibly elsewhere in Central Asia) would benefit from mass drug administration (MDA), possibly with a single dose of mebendazole, which is highly effective against both ascariasis and enterobiasis [17], in addition to health education programs and increased clean water availability [12].

## Zoonotic Helminth Infections

The major Central Asian zoonotic helminth infections include echinococcosis, opisthorchiasis, and fascioliasis; toxocariasis and trichinellosis are also present.

### Echinococcosis

A rise in the number of cases of cystic echinococcosis caused by *Echinococcus granulosus* has been noted since 1991 [8], [18], which Torgerson et al. attribute to the breakdowns in veterinary public health as outlined above [13]. Overall, cystic echinococcosis has increased 4- to 5-fold over the last two decades in four of the Central Asian nations and in parts of Kazakhstan [13]–[15]. Today, the incidence rates of infection approach 30 cases per 100,000 in some districts, although the disease is believed to be vastly underreported [13], [19]. Some areas may be considered hyperendemic—for instance, in a study in rural eastern Kazakhstan, approximately 1% of the population exhibited evidence of cystic echinococcosis by ultrasound [15]. Another concerning trend is the disproportionate rise in pediatric cases [13]. Such observations are complemented by veterinary epidemiologic studies in dogs showing that 20% or more of dogs harbor adult *E. granulosus* tapeworms [20], with a high prevalence in both Kyrgyzstan and Kazakhstan [20], [21]. Russia has also experienced an increase in imported cases from Central Asia [22]. Alveolar echinococcosis is also prevalent both among dogs, rodents, and in definitive host red foxes [20], [23]–[25].

### Fluke Infections: Opisthorchiasis and Fascioliasis

Globally, there are an estimated 1.2 million cases of opisthorchiasis caused by the liver fluke *Opisthrochis felineus*, which occur primarily in Western Siberia (the Ob and Irtysh River valleys), Ukraine, Belarus, and Kazakhstan [26]–. In Kazakhstan, opisthorchiasis is endemic in Aktyubinsk, Dzhezkazgan, Karaganda, Pavlodar, Tselinograd, and Turgay districts [26]. The infection is transmitted by the ingestion of uncooked and sometimes salted fish known as *stroganina* [26], and *O. felineus* has been associated with co-infection with *Helicobacter pylori* [29]. *O. felineus* infection results in fever and hepatitis, leading to abdominal pain. Advanced cases also result in suppurative cholangitis, liver fibrosis, and ultimately cholangiocarcinoma. An estimated 400 cases of cholangiocarcinoma occur annually from chronic *O. felineus* infection [30]. Fascioliasis and dicrocoeliasis are also found in Central Asia, with an overall approximate prevalence of close to 2% among school-aged children in Kyrgyzstan [12].

### Toxocariasis and Trichinellosis

Toxocariasis is prevalent in rural Kazakhstan, especially among children [15], and the infection may be common elsewhere in Central Asia. After *Trichinella spiralis, Trichinella britovi* is the second most common *Trichinella* species affecting humans and an important species in Central Asia [31]. *T. britovi* has a sylvatic life cycle with boars, horses, foxes, and jackals as animal reservoir hosts, although it can also infect domestic pigs [31]. *Trichinella pseudospiralis* is also prevalent [31]. There are no disease burden estimates for these species.

Pozio recently made recommendations to the European Union for the control of food-borne helminth infections [32], but they also apply to Central Asia. These recommendations include improvement of farming conditions with health education of livestock producers and farmers, increased efforts and improved methods to detect parasites in slaughtered animals, reductions in contact between livestock and wild animals, and control of sewage sludge on pastures [32].

## Vector-Borne Protozoan Infections

The two major protozoan infections in Central Asia are leishmaniasis and vivax malaria.

### Leishmaniasis

The two major forms of leishmaniasis are zoonotic cutaneous leishmaniasis (ZCL) and visceral leishmaniasis (VL). In Central Asia, ZCL is caused predominantly by *Leishmania major*, first described in 1914 by the Russian physicians Yakimov and Schokhor as *L. tropica major* from a patient in Uzbekistan [33]. Additional molecular typing has differentiated human *L. major* species (*L. major sensu stricto*) from the closely related animal species *Leishmania turanica* and *Leishmania gerbili* [33]. Human ZCL occurs when humans enter sylvatic habitats in river valleys that interrupt the deserts and piedmont plains of Central Asia where both the great gerbil, *Rhombomys optimus* [34], and the *Phlebotomus papatasi* sandfly vector [33], [35] are found. In Uzbekistan, a highly aggressive form of ZCL from *L. major* has also been reported [36]. There are no prevalence estimates available for *L. major* ZCL in Central Asia. *Leishmania infantum* is the major cause of VL in Uzbekistan and Tajikistan. This strain of *L. infantum* is believed to be separate from a strain found in Europe, the Middle East, and North Africa [37].

### Malaria

There is no endemic malaria in Kazakhstan, and in 2010 Turkmenistan became the first Central Asian country to become certified as free of endemic malaria following control efforts that date back to the 1920s [38]. Since the 1950s, only *Plasmodium vivax* has been present, with the last autochthonous case registered in 2004 [38]. Uzbekistan also reported zero cases for the first time in 2009 [39]. In contrast, Tajikstan and Kyrgyzstan still report endemic malaria [39]. In Tajikistan, 30% of the population was at risk for malaria in 2009 [39]; however, the number of cases is down considerably from a peak of 30,000 reported cases in 1997 [40] as a result of programs of indoor residual spraying and MDA of primaquine and other anti-malaria drugs in the years following a five-year civil conflict from 1992 to 1997 [40]–[42]. Almost all of the cases are a result of *P. vivax* infections that peak in August and September, and are transmitted primarily by *Anopheles superpictus* and *Anopheles pulcherrimus* in areas of cotton and rice field irrigation [40]. Children are disproportionately affected [43], [44], and the highest morbidity occurs in the southern Khation region that borders on Afghanistan [43]. The *P. vivax* epidemic there is very much fueled by human migrations from Afghanistan and a permissive climate [41]. In turn, human migrations from Tajikistan thwart efforts to eliminate malaria in Uzbekistan [45]. *P. vivax* is also the predominant malaria parasite in Kyrgyzstan [46]. Ultimately, control and elimination efforts for *P. vivax* malaria infections in Central Asia will depend upon ongoing indoor residual spraying with insecticides and MDA with primaquine (except for patients with glucose 6 phosphate dehydrogenase deficiency [40]–[42]). Such large-scale efforts are being supported by the Global Fund to Fight AIDS, Tuberculosis and Malaria [43]. Success in eliminating malaria will also depend on international cooperation with Afghanistan [47].

Among the other vector-borne neglected infections that exist in Central Asia are plague, tularemia, tick-borne relapsing fever, Crimean hemorrhagic fever, tick typhus, and Q fever [48].

## Other NTDs

Toxoplasmosis was noted to be prevalent in rural Kazakhstan [15], but the overall prevalence in the region is unknown. Brucellosis is an important bacterial NTD [49]. Similarly, intestinal protozoan infections such as amoebiasis and cryptosporidiosis are common and represent opportunistic infections among patients with HIV/AIDS [50]. Canine rabies is still present in the region [51].

## Concluding Remarks

With some exceptions, such as the detailed knowledge on malaria in the region [38], [39] and cystic echinococcosis [13], [15], there is an absence of baseline information on the prevalence and disease burden of Central Asia's NTDs in the English scientific literature. The dearth of information may reflect a general absence of surveillance efforts in the two decades following the breakup of the Soviet Union, in parallel with breakdowns in human and veterinary public health infrastructure in these countries, although our observation may also reflect the fact that much of this information may be contained in the Russian scientific literature, which we did not tap. Among the priorities are stepped-up surveillance activities for the major intestinal helminth infections, including ascariasis and enterobiasis, the major food-borne and zoonotic helminth infections, and leishmaniasis and other vector-borne NTDs. Also of importance would be efforts to learn more about non-vector-borne protozoan infections such as toxoplasmosis and intestinal infections, brucellosis, and rabies. Some of these activities would be greatly aided by increased international cooperation between the Central Asian republics and with Afghanistan, which could also launch efforts for MDA of affected populations with soil-transmitted helminth infections and malaria, and livestock and vector control for the zoonotic NTDs, and possibly the establishment of a new international research center for NTDs. The Global Fund to Fight AIDS, Tuberculosis, and Malaria might also consider how to best integrate NTDs into their control and elimination programs. The burden of NTDs appears to be high among the poorest people living in Central Asia, and there is an urgent need to tackle this problem using multi-dimensional approaches.
